# STEMI in Times of Crisis: Comparative Analysis During Pandemic and War

**DOI:** 10.3390/jcm14051720

**Published:** 2025-03-04

**Authors:** Vladimir Zeldetz, Sagi Shashar, Carlos Cafri, David Shamia, Tzachi Slutsky, Naif Abu Abed, Dan Schwarzfuchs

**Affiliations:** 1 Department of Emergency Medicine, Soroka University Medical Center, Beer-Sheva 84101, Israel; vladimirz@clalit.org.il (V.Z.); tzachisl@clalit.org.il (T.S.); naifab@clalit.org.il (N.A.A.); dansch@clalit.org.il (D.S.); 2 Clinical Research Center, Soroka University Medical Center, Faculty of Health Sciences, Ben-Gurion University of the Negev, Beer-Sheva 84101, Israel; 3 Department of Cardiology, Soroka University Medical Centre, Beer Sheva 84101, Israel; cafricar@clalit.org.il (C.C.); shamiada@clalit.org.il (D.S.); 4 Hospital Administration, Soroka University Medical Center, Beer Sheva 84101, Israel

**Keywords:** STEMI, COVID-19, war, mortality, healthcare delivery, crisis management

## Abstract

**Background:** Crises such as pandemics and wars significantly impact cardiovascular health, particularly ST-Elevation Myocardial Infarction (STEMI). The COVID-19 pandemic led to overwhelmed healthcare systems and delayed care, while the October 2023 war in Israel posed unique challenges, including altered patient behavior and access to care. This study compares STEMI outcomes during these two crisis periods, focusing on mortality and care pathways. **Methods:** This retrospective cohort study, conducted at Soroka University Medical Center, analyzed patients with STEMI during the COVID-19 lockdown (14 March 2020–14 June 2020), the war period (07 October 2023–7 January 2024), and quiet periods in 2022. Patient demographics, arrival methods, and outcomes were compared. Multivariable logistic regression identified mortality predictors. **Results:** Among 397 patients with STEMI, 30-day mortality was 7.5 times higher during COVID-19 (OR 7.50, *p* = 0.038), and in-hospital mortality was 10.25 times higher (OR 10.25, *p* = 0.046) compared to the war. The war period showed an 86% reduction in 30-day mortality (OR 0.14, *p* = 0.026). More patients arrived by ambulance during COVID-19, while during the war, more were referred via emergency medical centers and admitted directly to the ICCU. **Conclusions:** The COVID-19 pandemic significantly increased STEMI mortality, while the war’s coordinated care pathways improved outcomes. Tailored crisis management strategies are important to ensure effective acute care during pandemics and conflicts.

## 1. Introduction

ST-Elevation Myocardial Infarction (STEMI) is a severe cardiovascular event, and its management is affected by external stressors such as disasters and prolonged crises [1,2]. During the COVID-19 pandemic, STEMI admissions declined, likely due to patients’ fear of infection, overwhelmed healthcare systems, and delayed treatment-seeking [3]. Logistical challenges, including limited catheterization lab availability and the reassignment of staff to COVID-19 care, contributed to worse outcomes, with prolonged door-to-balloon (D2B) times reported in many regions [4].

In contrast, the impact of war on STEMI remains less studied. Wars present distinct challenges, including physical danger, disrupted healthcare services, and heightened psychological stress among both patients and healthcare providers [2]. Limited studies suggest that STEMI incidence may increase during wars, potentially due to these stressors and difficulties in accessing timely care [5]. However, existing studies are few, often focused on specific regional conflicts, and lack comprehensive data on the management of STEMI in such settings.

Similarly, natural disasters and terrorist attacks have been linked to increased STEMI incidence, often associated with acute stress responses in susceptible individuals [6,7]. Similarly, natural disasters and terrorist attacks have been linked to increased STEMI incidence, often associated with acute stress responses in susceptible individuals [8]. Moreover, the mechanisms underlying these crises’ cardiovascular effects and optimal management strategies remain unclear [9].

This study investigates how two prolonged crises—COVID-19 and war—impact STEMI incidence, management, and outcomes. We hypothesize that crisis type influences STEMI outcomes due to differences in healthcare system strain, resource allocation, and patient behavior. We anticipated higher STEMI mortality during COVID-19 due to delayed care and systemic overload, whereas war-related STEMI management may be more efficient due to streamlined emergency pathways. By comparing these crises with quiet periods, this study aims to identify crisis-specific strategies to optimize STEMI care, ultimately improving patient outcomes and healthcare system resilience in future emergencies.

## 2. Methods

### 2.1. Study Design

This retrospective cohort study was conducted at Soroka University Medical Center (SUMC) in Beer Sheva, Israel, the primary tertiary care hospital serving the southern region of Israel, including the Negev. The study analyzed three key periods: the initial COVID-19 lockdown (14 March–14 June 2020), the war period (7 October 2023–7 January 2024), and two quiet periods in 2022 (14 March–14 June and 7 October–7 January). This design accounts for seasonal variations in STEMI incidence, as cardiovascular events are known to fluctuate based on environmental and healthcare system factors [10,11]. Comparing each crisis to its corresponding control period ensures that observed differences reflect the impact of the crisis rather than natural fluctuations in STEMI occurrence [12].

### 2.2. Crisis Periods

These periods were selected to compare the impact of different prolonged stressors—namely, a global pandemic and an active military conflict—on the management and outcomes of STEMI. The initial COVID-19 lockdown was characterized by stringent nationwide restrictions, including severe limitations on movement, the closure of non-essential services, and significant reallocation of healthcare resources to manage the pandemic. These measures, while necessary to control the spread of the virus, resulted in disruptions to routine medical care, including the treatment of acute cardiovascular conditions such as STEMI.

The war period began on 7 October 2023, with intense rocket fire from Gaza targeting southern Israel, including Beer Sheva. The Negev region became a war zone, with frequent air raid sirens and missile strikes, heightening stress for both civilians and the healthcare system. During this time, SUMC played a critical role in providing emergency medical services to the citizens in the region and to injured soldiers, managing the dual challenges of treating war-related injuries and continuing routine and acute care under the constant threat of rocket attacks.

### 2.3. Ethics

The study was approved by the institutional Helsinki review board (approval number: 0298-23-SOR). Given the retrospective nature of the study, informed consent was waived. Data were carefully extracted from the institutional database, ensuring that all patient information was anonymized and encoded to maintain confidentiality.

### 2.4. Study Population

The study included all adult patients (aged 18 years and older) diagnosed with ST-elevation myocardial infarction (STEMI) during the specified periods and admitted either to the cardiac care unit for urgent catheterization or to the emergency department. The analysis included patients who presented within 12 h of symptom onset, as well as those who arrived between 12 and 48 h after symptom onset but required urgent intervention due to clinical instability, such as ongoing chest pain with ST elevation on ECG, hemodynamic instability, or significant arrhythmias. This approach aligns with the 2017 ESC Guidelines [13], which recommend PCI for patients presenting within 12 h and consider PCI for those presenting between 12 and 48 h with clinical indications. Patients presenting more than 48 h after symptom onset without indications for urgent intervention were excluded, as routine PCI in this context is not recommended by the guidelines. Additional exclusions were patients under 18 years old, those transferred from other hospitals, and non-residents of Israel.

### 2.5. Data Collection

Data were extracted from the computerized medical records at SUMC. Collected variables included demographic information (age, gender, ethnicity), medical history (including ischemic heart disease, hypertension, diabetes, and smoking status), and the Charlson Weighted Comorbidity Index (CWCI). Timing metrics recorded included the interval from symptom onset to first medical contact, hospital arrival, the start of catheterization, and artery opening, alongside the total ischemic time. Clinical data also included the presence of cardiac arrhythmias, cardiogenic shock, and left ventricular function, which was assessed via echocardiography within 48 h of admission.

### 2.6. Outcomes

The primary outcomes of the study were in-hospital mortality and 30-day mortality.

### 2.7. Statistical Analysis

Categorical variables were described as frequencies and percentages, while continuous variables were summarized as means with standard deviations (SD) for normally distributed data or medians with interquartile ranges (IQR) for non-normally distributed data. Comparative analyses were conducted to assess differences between the COVID-19 period and the corresponding period in 2022, the war period and its corresponding period in 2022, and directly between the COVID-19 and war periods. Categorical variables were compared using the Chi-squared test, while continuous variables were analyzed with the unpaired Student’s t-test for parametric data and the Mann–Whitney U test for non-parametric data. Survival analysis was performed using the Kaplan–Meier method to compare 30-day mortality among the four groups: COVID-19, non-COVID, war, and non-war periods. Log-rank tests were used to assess statistical differences between survival curves. Pairwise comparisons were conducted for three key contrasts: COVID vs. non-COVID, war vs. non-war, and COVID vs. war, with corresponding *p*-values reported. Survival probabilities over time were graphically represented using Kaplan–Meier curves, with the number of patients at risk displayed at different time points (0, 10, 20, and 30 days).

Univariable and multivariable logistic regression models were utilized to explore associations between the study periods and clinical outcomes. The selection of variables for the multivariable models was based on prior research, clinical relevance, and findings from the univariable analysis. Results were reported as adjusted odds ratios (AdjORs) with 95% confidence intervals (95% CIs), and statistical significance was set at a *p*-value of less than 0.05. All statistical analyses were conducted using RStudio version 2024.04.2.

### 2.8. Sensitivity Analysis

To assess the robustness of our findings, we conducted a sensitivity analysis comparing logistic regression and Cox proportional hazards models for 30-day mortality. Model selection was based on stepwise AIC optimization and likelihood ratio testing. While stepwise selection identified a more parsimonious model, the difference in AIC values between the models was minimal. Given the complexity of STEMI outcomes and the need to adjust for multiple clinical and logistical factors, we chose to retain the full logistic regression model to ensure maximal adjustment for potential confounders. The logistic regression model also demonstrated superior fit and predictive performance compared to the Cox model, leading to its selection as the primary model for analysis.

### 2.9. AI Assistance

We used ChatGPT-4, an AI technology by OpenAI, to assist in drafting and refining the text of this manuscript for clarity and coherence. All AI-generated content was reviewed and edited by the authors to ensure accuracy and relevance

## 3. Results

A total of 397 patients diagnosed with STEMI were included in the study, with 99 patients from the COVID-19 period, 102 from the corresponding non-COVID period in 2022, 97 from the war period, and 99 from the corresponding non-war period in 2022. The demographic and clinical characteristics, as well as outcomes of these patients, were compared across the different periods and are shown in Table 1.

The mean age of the overall cohort was 60.78 years (SD 12.75). No significant difference in age was observed between the COVID-19 and non-COVID periods (*p* = 0.713); however, patients during the war period were significantly younger than those in the non-war period (mean age 59.96 vs. 65.18 years, *p* = 0.003). Gender distribution was similar across all periods, with no significant differences observed.

Comorbidities, including diabetes mellitus, hypertension, dyslipidemia, and smoking status, did not show significant variation between the periods. The Charlson Comorbidity Index (CCI) was similar between the COVID-19 and non-COVID periods (median [range] 2.00 [0.00–9.00] vs. 2.00 [0.00–7.00], *p* = 0.676) but was significantly higher in the non-war period compared to the war period (median [range] 3.00 [0.00–7.00] vs. 2.00 [0.00–7.00], *p* = 0.024).

**Figure 1:**
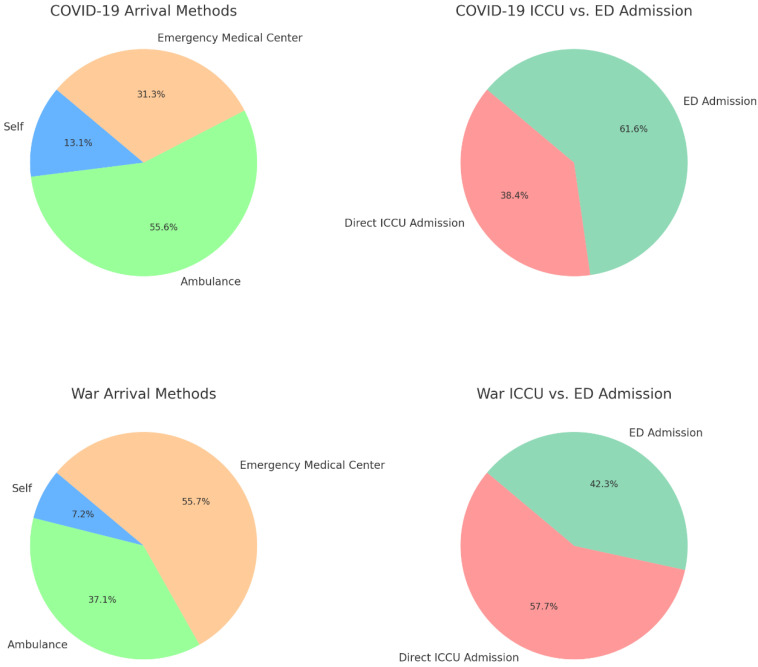
Comparison of arrival methods and admissions during COVID-19 and war periods. Figure 1 visually compares the distribution of arrival methods and admission types for patients with STEMI during the two crisis periods: COVID-19 and War. Each pie chart segment represents the percentage of patients who used a particular method of arrival (self, ambulance, emergency medical center) or were admitted directly to the ICCU versus through the ED. The top row of charts displays data for the COVID-19 period, with the left chart showing the distribution of arrival methods and the right chart focusing on the admission types (ICCU vs. ED). The bottom row presents the same information for the war period. The consistent color scheme across the arrival method charts helps to easily compare the patterns between the two periods, while the distinct colors used in the ICCU vs. ED admission charts emphasize the differences in patient flow during these crises.

In terms of arrival time, a higher percentage of patients arrived within 12 h during the non-war period compared to the war period (92.9% vs. 77.3%, *p* = 0.004), and similarly, a significant difference was observed between the COVID-19 and war periods (87.9% vs. 77.3%, *p* = 0.028). The mode of arrival showed differences as well, with more patients arriving by ambulance during the COVID-19 period compared to the non-COVID period (55.6% vs. 39.2%, *p* = 0.056) (Figure 1). During the war period, a significantly higher proportion of patients were referred by an emergency medical center compared to the non-war period (55.7% vs. 35.4%, *p* = 0.008) and the COVID-19 period (55.7% vs. 31.3%, *p* = 0.003) (Figure 1). Additionally, more patients were admitted directly to the intensive cardiac care unit (ICCU) during the war period compared to the COVID-19 period (57.7% vs. 38.4%, *p* = 0.01), Figure 1.

Regarding complications, there were no significant differences in LVF across periods. However, the median D2B time was significantly shorter during the war period (48 vs. 70 min, *p* = 0.045) compared to the non-war period but showed no significant difference between the COVID-19 and non-COVID periods (60 vs. 55 min, *p* = 0.642). Additionally, spontaneous reperfusion was more frequent during the COVID-19 period compared to the war period (22.2% vs. 9.3%, *p* = 0.022).

**Figure 2:**
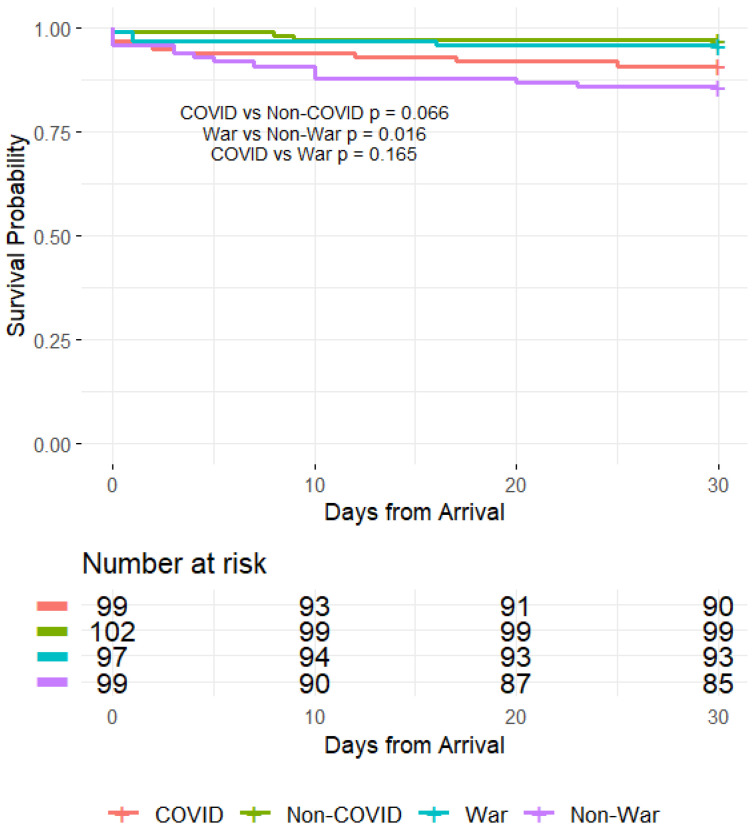
Survival analysis of patients with STEMI during COVID-19 and war periods. Figure 2 presents Kaplan–Meier survival curves comparing 30-day mortality among patients with STEMI across four crisis periods: COVID-19 (red), non-COVID (green), war (blue), and non-war (purple). The *x*-axis represents days from hospital arrival, while the *y*-axis denotes survival probability. The risk table below the plot displays the number of patients at risk at different time points (0, 10, 20, and 30 days).

In-hospital mortality rates were higher during the COVID-19 period compared to the non-COVID period, though not significantly (7.1% vs. 2.9%, *p* = 0.307). Notably, 30-day mortality was significantly higher during the non-war period compared to the war period (14.1% vs. 4.1%, *p* = 0.029). Survival rates were lower during the COVID-19 period than in the non-COVID period, with a trend toward statistical significance (*p* = 0.066). A significant difference in survival was observed between the war and non-war periods (*p* = 0.016), indicating improved survival outcomes during the war period. However, no statistically significant difference was found between the COVID-19 and war periods (*p* = 0.165), Figure 2. Length of stay in the ICCU did not differ significantly across the periods.

**Figure 3:**
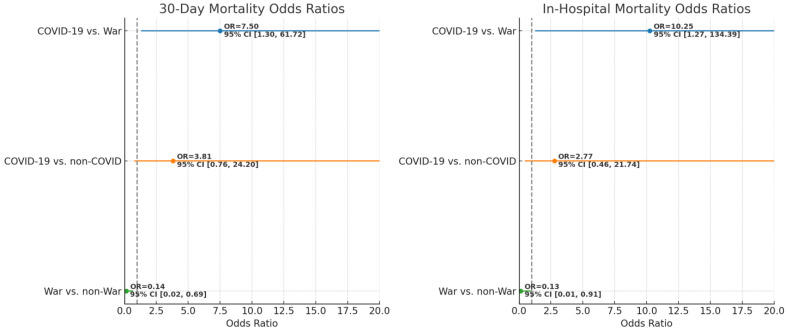
Comparison of 30-day and in-hospital mortality odds ratios across COVID-19, war, and non-crisis. The forest plot visually compares the odds ratios for 30-day and in-hospital mortality across the crisis periods: COVID-19, war, and non-COVID/non-war periods. Each point on the plot represents an odds ratio, with the horizontal lines extending from the points indicating the 95% confidence intervals. The vertical dashed line at 1.0 represents the null hypothesis, where no difference in odds exists between the groups being compared. The left panel displays the odds ratios for 30-day mortality, while the right panel focuses on in-hospital mortality. In both panels, comparisons between COVID-19 vs. war, COVID-19 vs. non-COVID, and war vs. non-war are shown. The use of a consistent color scheme helps distinguish between different comparisons, with each color corresponding to a specific pair of groups being compared.

In the logistic regression analysis shown in Table 2 and illustrated in Figure 3, after adjusting for gender, age, nationality, comorbidities, mode of arrival, left ventricular function, length of stay in the ICCU, and the presence of cardiogenic shock prior to PCI, significant differences were observed between the groups regarding both 30-day and in-hospital mortality. Specifically, patients in the COVID-19 period had significantly higher odds of 30-day mortality compared to those in the war period (OR = 7.50, 95%CI [1.30–61.72], *p* = 0.038), and similarly higher odds of in-hospital mortality (OR = 10.25, 95%CI [1.27–134.39], *p* = 0.046). Conversely, the war period was associated with significantly lower odds of 30-day mortality compared to the non-war period (OR = 0.14, 95%CI [0.02–0.69], *p* = 0.026), with a similar trend observed for in-hospital mortality, although the latter was marginally non-significant (OR = 0.13, 95%CI [0.01–0.91], *p* = 0.058). There were no statistically significant differences in mortality between the COVID-19 and non-COVID periods.

## 4. Discussion

This study revealed several key findings about STEMI outcomes during different crisis periods. Both 30-day and in-hospital mortality rates were significantly higher during the COVID-19 period, with 30-day mortality 7.5 times higher and in-hospital mortality 10.25 times higher than during the war period. Conversely, the war period saw an 86% reduction in 30-day mortality compared to the non-war period. Patients during the war were younger and had a lower CCI. During COVID-19, more patients arrived by ambulance, while during the war, a higher proportion were referred by emergency medical centers. Additionally, more patients were admitted directly to the ICCU during the war compared to both the COVID-19 and non-war periods.

### 4.1. Explanations for Higher Mortality in COVID-19 vs. War

The increased mortality during COVID-19 compared to the war period may be linked to COVID-19’s prothrombotic effects, which differ from the usual plaque rupture mechanism of STEMI. Studies suggest that COVID-19-associated STEMI often results from arterial thrombosis and microvascular injury, leading to worse myocardial damage and reduced effectiveness of reperfusion therapy [14]. Additionally, many patients had concurrent SARS-CoV-2 infection, exacerbating their condition through endothelial dysfunction, cytokine storm, and systemic inflammation [15]. Healthcare system strain during COVID-19, including ICU overload and delayed STEMI treatment, likely worsened outcomes [16]. In contrast, patients with STEMI during the war faced logistical challenges but were not simultaneously battling a systemic illness, which may explain their lower mortality despite crisis conditions. These findings emphasize the need for crisis-specific STEMI management strategies tailored to different pathophysiological mechanisms [17,18].

### 4.2. Differences in Healthcare Access During Crises

During COVID-19, more patients arrived first at the ED, likely seeking comprehensive care for what they perceived as severe illness. In contrast, during the war period, a higher proportion of patients were referred by emergency medical centers, likely due to fear of traveling to a hospital under threat of rocket attacks and concerns about entering a busy and potentially chaotic hospital environment. The war also saw more patients admitted directly to the ICCU, bypassing the ED, which may have facilitated quicker and more efficient treatment. These differences suggest that the war period allowed for more direct and coordinated care. The immediate threat of rocket attacks and the overall atmosphere of heightened alertness likely led to a more streamlined approach to emergency care, where local emergency medical centers became crucial points of initial contact [19]. This enabled patients to receive timely care closer to home, avoiding the dangers of traveling through exposed areas. Additionally, the need for rapid triage and treatment under the constant threat of further attacks may have prompted faster decision-making and more efficient use of resources, such as direct admissions to the ICCU [20]. This heightened focus on rapid and effective care during the war likely played a significant role in improving survival rates, despite the challenging circumstances.

### 4.3. Controversial Role of Smoking in STEMI Outcomes

Smoking is a well-known risk factor for cardiovascular disease, but paradoxically, some studies suggest it may be associated with better short-term outcomes in patients with STEMI, known as the “smoker’s paradox” [21]. This may relate to differences in baseline characteristics, such as younger age and fewer comorbidities among smokers. However, the long-term outcomes of smoking, particularly during stressors like pandemics and wars, should be further investigated.

### 4.4. Comparison of War and Non-War Periods

The lower mortality observed during the war period compared to the non-war period is likely due to a combination of factors. Patients admitted during the war were younger and had low CCI scores, suggesting a relatively healthier and more resilient population. Additionally, D2B times were shorter, reflecting more efficient healthcare pathways and reduced treatment delays, likely due to heightened alertness and improved crisis response protocols [22]. A key aspect of this efficiency was the shift in healthcare access. More patients were referred by emergency medical centers rather than self-presenting to the ED, facilitating earlier stabilization and reducing prehospital delays. In contrast, during non-war periods, higher rates of self-referrals contributed to longer delays before definitive care. Direct admissions to the ICCU were also more frequent during the war, ensuring that critically ill patients bypassed the ED and received immediate specialized care [5].

Unlike the COVID-19 period, where hospitals faced widespread systemic strain, the war period saw targeted resource allocation, prioritizing emergency cases. This crisis-induced restructuring likely contributed to more streamlined STEMI management. The combination of a younger patient population, direct ICCU admissions, reduced treatment delays, and a well-coordinated crisis response—including heightened alertness and resource availability—enabled rapid and effective interventions despite the ongoing conflict [23].

### 4.5. Comparison with Existing Literature

When examining STEMI during COVID-19, our findings are consistent with other studies that reported increased mortality and delayed care for cardiovascular events during the pandemic [24]. The strict lockdowns, fear of infection, and overwhelmed healthcare systems globally led to decreased healthcare utilization, delayed presentations, and ultimately poorer outcomes for patients with STEMI [25]. However, the degree of impact varied across different regions and healthcare systems, with some studies reporting even more severe delays and higher mortality rates than those observed in our study [26].

Similarly, STEMI outcomes during wartime have been less frequently studied, but the available literature suggests that wars generally increase cardiovascular events due to heightened stress and care disruptions [2,5]. However, our finding of lower mortality during the war contrasts with some reports, likely due to the unique context of this study. The war in Gaza, which directly affected the Negev region, prompted a robust and immediate response from healthcare services at SUMC. The proximity of the conflict led to changes in healthcare utilization, with more patients choosing local emergency centers over the hospital due to safety concerns. Additionally, the effective triage system at SUMC “physician in triage” [27], where physicians rapidly assessed and prioritized acute cases like STEMI, ensured that critically ill patients were quickly routed to the ICCU or catheterization lab. This streamlined approach, combined with direct admissions to the ICCU, likely contributed to the improved outcomes observed during the war, contrasting with the higher mortality rates typically associated with such conflicts.

### 4.6. Implications for Disaster Preparedness

During the war period, the use of local emergency medical centers and the direct routing of patients to the ICCU at SUMC facilitated faster treatment and likely contributed to lower mortality rates. This highlights the importance of protocols that prioritize rapid triage and direct admission to specialized care units during crises. Conversely, during the COVID-19 pandemic, the overloaded ICUs and patients’ preference for arriving at the ED underscored the need for clear communication strategies that guide patients to the safest and most effective care settings. Public health interventions should focus on ensuring that patients receive timely care, whether through ambulance services or referrals to less congested facilities, depending on the nature of the crisis. The structured response observed in the war period suggests that pre-defined crisis pathways can mitigate delays and optimize outcomes, reinforcing the importance of proactive disaster planning for cardiovascular emergencies [28]. These findings highlight the need for tailored strategies to maintain effective acute care services during crises. Future research should aim to quantify the specific impact of coordinated triage and direct hospital admission models in crisis settings, facilitating the development of adaptable frameworks for both pandemic and wartime emergencies [29].

### 4.7. Limitations

This study has several limitations. As a retrospective cohort study conducted at a single tertiary care center, the findings may not be fully generalizable to other regions or healthcare systems with different infrastructures, crisis response protocols, or patient demographics. The small sample size further limits the strength of conclusions, though SUMC remains the only tertiary referral center in the Negev, serving a highly diverse population. The unique context of the war-affected Negev region and the COVID-19 lockdown conditions in Israel may not fully reflect STEMI outcomes in other crises. However, the inclusion of both urban and rural populations, including diverse ethnic groups, ensures that the findings are relevant to populations with similar healthcare challenges. Despite this, healthcare delivery models and resource availability during crises may differ significantly across countries and healthcare settings. Therefore, future studies from different geographical regions and healthcare systems are needed to validate these findings and assess their applicability across various healthcare models.

A key limitation of this study is the lack of prehospital mortality data. Both COVID-19 and war-related crises may have led to an increase in out-of-hospital deaths, given the disruptions in emergency medical services and potential delays in seeking care. As our study included only patients who arrived at the hospital, the true burden of STEMI mortality during crises may be underestimated. Future research should incorporate prehospital STEMI mortality data to capture the full impact of these crises on cardiovascular outcomes.

Additionally, while we adjusted for multiple confounders, unmeasured factors such as the severity of COVID-19 infections, the impact of war on healthcare infrastructure, and psychological stress on both patients and healthcare providers may have influenced the outcomes. Moreover, factors such as socioeconomic status, financial barriers, and disparities in healthcare access—which could have affected STEMI outcomes—were not accounted for due to data limitations. Furthermore, our study focused on all-cause mortality without distinguishing between deaths directly attributable to STEMI and those from other complications. Future multi-center studies incorporating prehospital data, socioeconomic indicators, and long-term follow-up will be crucial in developing comprehensive crisis preparedness strategies for STEMI management and in better understanding STEMI-specific mortality patterns during crises.

## 5. Conclusions

This study reveals that COVID-19 led to significantly higher STEMI mortality compared to war, with a 7.5 times greater risk of 30-day mortality, likely due to increased coagulability and overwhelmed ICUs. In contrast, the war period’s more direct and coordinated care pathways resulted in better outcomes, with an 86% reduction in mortality, likely due to the younger patient population, lower CCI, and the efficiency of the more direct and coordinated care pathways, despite the challenges of the conflict. These findings highlight the importance of tailored crisis management strategies in ensuring timely and effective acute care during different types of crises, though further research is needed to optimize these approaches and assess their applicability across various healthcare settings.

## Figures and Tables

**Table 1:** Baseline characteristics, comorbidities, arrival characteristics, timelines, complications, and outcomes among patients with STEMI across different crisis periods (COVID-19, war, non-war, non-COVID). The table displays a comparison of demographic and clinical characteristics across different crisis periods: COVID-19, war, non-war, and non-COVID periods, with a *p*-value of <0.05 considered statistically significant.

	Overall	COVID	Non-COVID	COVID vs. No COVID *p*-Value	War	Non-War	WAR vs. No War *p*-Value	COVID vs. War *p*-Value
**Demographics**	397	99	102		97	99		
Female sex (*n*, %)	67 (16.9)	18 (18.2)	14 (13.7)	0.503	14 (14.4)	21 (21.2)	0.293	0.605
Age, years (mean ± SD)	60.78 (12.75)	58.68 (12.71)	59.34 (12.91)	0.713	59.96 (12.36)	65.18 (12.13)	**0.003**	0.475
Bedouin nationality (*n*, %)	125 (31.5)	31 (31.3)	37 (36.3)	0.552	28 (28.9)	29 (29.3)	1	0.828
**Comorbidities**								
CIHD (*n*, %)	85 (21.4)	18 (18.2)	21 (20.6)	0.8	27 (27.8)	19 (19.2)	0.208	0.151
Diabetes mellitus (*n*, %)	148 (37.3)	30 (30.3)	35 (34.3)	0.648	40 (41.2)	43 (43.4)	0.868	0.148
Hypertension (*n*, %)	189 (47.6)	46 (46.5)	44 (43.1)	0.74	54 (55.7)	45 (45.5)	0.198	0.252
Dyslipidemia (*n*, %)	311 (78.3)	73 (73.7)	75 (73.5)	1	83 (85.6)	80 (80.8)	0.484	0.06
Smoking (*n*, %)	217 (54.7)	54 (54.5)	61 (59.8)	0.541	53 (54.6)	49 (49.5)	0.563	1
CCWI (median [IQR])	2.00 [0.00, 9.00]	2.00 [0.00, 9.00]	2.00 [0.00, 7.00]	0.676	2.00 [0.00, 7.00]	3.00 [0.00, 7.00]	0.024	0.752
**Arrival characteristics**								
Arrival time (%)				0.221			**0.004**	**0.028**
Above 48 h	27 (6.8)	9 (9.1)	4 (3.9)		9 (9.3)	5 (5.1)		
Between 12–48 h	24 (6.0)	3 (3.0)	6 (5.9)		13 (13.4)	2 (2.0)		
Within 12 h	346 (87.2)	87 (87.9)	92 (90.2)		75 (77.3)	92 (92.9)		
Arrival by, *n* (%)				**0.056**			**0.008**	**0.003**
Self	59 (14.9)	13 (13.1)	22 (21.6)		7 (7.2)	17 (17.2)		
Ambulance ordered by patient	178 (44.8)	55 (55.6)	40 (39.2)		36 (37.1)	47 (47.5)		
Referral by an emergency medical center	160 (40.3)	31 (31.3)	40 (39.2)		54 (55.7)	35 (35.4)		
Arrival directly ICCU vs. ED, *n* (%)	189 (47.6)	38 (38.4)	45 (44.1)	0.495	56 (57.7)	50 (50.5)	0.383	**0.01**
**Timelines (minutes)**								
Pain to first medical contact (median [IQR])	97.50 [10.00, 690.00]	90.00 [10.00, 690.00]	92.50 [10.00, 690.00]	0.918	90.00 [15.00, 620.00]	120.00 [10.00, 650.00]	0.818	0.317
Pain to door (median [IQR])	142.00 [25.00, 715.00]	120.00 [25.00, 715.00]	130.00 [30.00, 710.00]	0.911	145.00 [40.00, 710.00]	165.00 [30.00, 690.00]	0.532	0.286
Door to device (median [IQR])	41.50 [5.00, 420.00]	45.00 [10.00, 270.00]	42.00 [5.00, 150.00]	0.367	35.00 [10.00, 360.00]	50.00 [10.00, 420.00]	0.093	0.074
Door to balloon (median [IQR])	60.00 [15.00, 435.00]	60.00 [20.00, 300.00]	55.00 [20.00, 165.00]	0.642	48.00 [20.00, 365.00]	70.00 [15.00, 435.00]	**0.045**	0.306
Total ischemic time (median [IQR])	200.00 [82.00, 785.00]	185.00 [90.00, 779.00]	189.00 [82.00, 785.00]	0.895	200.00 [88.00, 525.00]	220.00 [85.00, 760.00]	0.513	0.263
**Complications**								
Left ventricular function (*n*, %)				0.504			0.193	0.737
Normal/preserved	66 (16.6)	15 (15.2)	17 (16.7)		18 (18.6)	16 (16.2)		
Mild dysfunction	136 (34.3)	33 (33.3)	41 (40.2)		33 (34.0)	29 (29.3)		
Moderate dysfunction	117 (29.5)	30 (30.3)	30 (29.4)		31 (32.0)	26 (26.3)		
Severe dysfunction	78 (19.6)	21 (21.2)	14 (13.7)		15 (15.5)	28 (28.3)		
Life-threatening arrhythmia (*n*, %)				0.759			0.076	0.577
No arrhythmia	341 (85.9)	86 (86.9)	92 (90.2)		83 (85.6)	80 (80.8)		
VT/VF	39 (9.8)	9 (9.1)	7 (6.9)		7 (7.2)	16 (16.2)		
High degree AV block	17 (4.3)	4 (4.0)	3 (2.9)		7 (7.2)	3 (3.0)		
Cardiogenic shock (*n*, %)	21 (5.3)	3 (3.0)	2 (2.0)	0.973	7 (7.2)	9 (9.1)	0.827	0.314
**Outcomes**								
In-hospital mortality (*n*, %)	23 (5.8)	7 (7.1)	3 (2.9)	0.307	3 (3.1)	10 (10.1)	0.092	0.347
30-day mortality (*n*, %)	30 (7.6)	9 (9.1)	3 (2.9)	0.123	4 (4.1)	14 (14.1)	0.029	0.267
LOS ICCU (median [IQR])	4.00 [1.00, 28.00]	4.00 [1.00, 28.00]	4.00 [1.00, 10.00]	0.794	4.00 [1.00, 16.00]	4.00 [1.00, 15.00]	0.538	0.277

STEMI: ST-Elevation Myocardial Infarction; ICCU: Intensive Cardiac Care Unit; CIHD: Chronic Ischemic Heart Disease; CCWI: Charlson Comorbidity Weighted Index; VT: Ventricular Tachycardia; VF: Ventricular Fibrillation; LOS: Length of Stay. Significant results (*p*-value < 0.05) are bolded.

**Table 2:** Logistic regression analysis of 30-day and in-hospital mortality across different crisis periods.

	30-Day Mortality			In-Hospital Mortality		
Predictors	Odds Ratios	CI	*p*-Value	Odds Ratios	CI	*p*
(Intercept)	0.00	0.00–0.00	**<0.001**	0.00	0.00–0.01	**0.001**
COVID vs. war	7.50	1.30–61.72	**0.038**	10.25	1.27–134.39	**0.046**
War vs. non-war	0.14	0.02–0.69	**0.026**	0.13	0.01–0.91	**0.058**
COVID vs. non-COVID	3.81	0.76–24.20	0.121	2.77	0.46–21.74	0.287
Female vs. male	1.43	0.41–4.71	0.560	0.72	0.14–3.21	0.680
Age	1.06	1.00–1.13	0.071	1.06	0.98–1.15	0.163
Bedouins vs. Jews	1.63	0.47–5.63	0.434	2.41	0.56–11.12	0.238
CCI	1.02	0.63–1.60	0.923	0.83	0.43–1.48	0.548
Arrival by ambulance vs. self	2.86	0.46–27.83	0.303	2.11	0.30–23.36	0.491
Arrival by emergency medical center vs. self	4.88	0.80–50.92	0.125	1.43	0.20–15.13	0.739
Arrival time between 12–48 h vs. above 48 h	0.00	NA	0.989	0.00	NA	0.993
Arrival time within 12 h vs. above 48 h	0.35	0.07–1.74	0.187	0.13	0.02–0.87	**0.034**
LVF	4.43	2.31–9.97	**<0.001**	5.81	2.43–19.47	**0.001**
LOS ICCU	1.00	0.81–1.17	0.969	0.92	0.72–1.13	0.523
Cardiogenic shock prior PCI	24.71	6.23–117.89	**<0.001**	54.65	11.09–387.86	**<0.001**

Logistic regression models were employed to assess the impact of different crisis periods—COVID-19, war, non-war, and non-COVID—on 30-day and in-hospital mortality among patients with STEMI. Odds ratios (ORs) with 95% confidence intervals (CIs) were calculated to compare the mortality outcomes across the different periods. Significant results (*p*-value < 0.05) are bolded. CI: Confidence Interval; STEMI: ST-Elevation Myocardial Infarction; ICCU: Intensive Cardiac Care Unit; PCI: Percutaneous Coronary Intervention; CCI: Charlson Comorbidity Index; LVF: Left Ventricular Function; NA: not available

## Data Availability

The research data supporting the results of this manuscript are not publicly available due to restrictions imposed by the institutional ethics committee. However, data may be made available upon reasonable request and subject to specific approval by the ethics committee. Requests for data access can be directed to the corresponding author and will be reviewed in accordance with the ethical guidelines and institutional policies.

## Abbreviations

STEMI	ST-Elevation Myocardial Infarction
PCI	Percutaneous Coronary Intervention
SUMC	Soroka University Medical Center
LVF	Left Ventricular Function
VT/VF	Ventricular Tachycardia/Ventricular Fibrillation
CCI	Charlson Comorbidity Index
CIHD	Chronic Ischemic Heart Disease
DM	Diabetes Mellitus
HTN	Hypertension
ED	Emergency Department
ICCU	Intensive Cardiac Care Unit
FMC	First Medical Contact
P2D	Pain to Door
D2N	Door to Needle
D2B	Door to Balloon
P2B	Pain to Balloon
AdjOR	Adjusted Odds Ratio
SD	Standard Deviation
IQR	Interquartile Range
LOS	Length of Stay
